# Causal association between common rheumatic diseases and glaucoma: a Mendelian randomization study

**DOI:** 10.3389/fimmu.2023.1227138

**Published:** 2023-09-19

**Authors:** Yang Meng, Zongbiao Tan, Yu Su, Lu Li, Changzheng Chen

**Affiliations:** ^1^ Department of Ophthalmology, Renmin Hospital of Wuhan University, Wuhan, China; ^2^ Department of Gastroenterology, Renmin Hospital of Wuhan University, Wuhan, China

**Keywords:** rheumatic diseases, Spondyloarthritis, glaucoma, genetics, single-nucleotide polymorphisms, Mendelian randomization

## Abstract

**Background:**

Autoimmunity and inflammation are the main characteristics of rheumatic diseases and have both been found to be related to glaucoma. However, it remains unclear whether rheumatic diseases increase the risk of glaucoma. Here, we performed a Mendelian randomization (MR) analysis to investigate the causal effects of six common rheumatic diseases on glaucoma.

**Methods:**

Six rheumatic diseases were included: ankylosing spondylitis (AS), rheumatoid arthritis (RA), systemic lupus erythematosus (SLE), Sicca syndrome/Sjögren’s sydrome (SS), dermatomyositis (DM), and gout. Glaucoma included primary open-angle glaucoma (POAG) and primary angle-closure glaucoma (PACG). Genetic variants associated with these rheumatic diseases and glaucoma were extracted from the genome-wide association studies and FinnGen8 database, respectively. First, a two-sample MR was used to investigate the potential causal association. Then, a multivariable MR was conducted to further verify the results. Inverse-variance weighted MR analysis was used as the main method, together with several sensitivity analyses.

**Results:**

Two-sample MR suggests that AS is related to a higher risk of both POAG [odds ratio (OR): 1.28, 95% confidence interval (CI) 1.13–1.44; *p* = 1.1 × 10^−4^] and PACG (OR: 1.55, 95% CI: 1.09–2.09, *p* = 1.4 × 10^−2^). Multivariable MR shows a similar trend of the effect of AS on POAG (OR: 1.52, 95% CI: 1.22–1.90, *p* = 1.9 × 10^−4^) and PACG (OR: 2.05, 95% CI: 1.06–3.95, *p* = 3.2 × 10^−2^). No significant association was observed between the other five rheumatic diseases and glaucoma.

**Conclusions:**

AS is related to an increased risk of POAG and PACG. We stress the importance of glaucoma screening for AS patients.

## 1 Introduction

Rheumatic diseases, including over 100 disorders, are a spectrum of autoimmune and/or inflammatory diseases that may damage the joints, muscles, bones, and organs (1). Rheumatic diseases are the most common cause of disability among US adults, surpassing heart diseases, diabetes, and cancers (2). As diseases with multi-organ involvement, rheumatic diseases can affect the brain, heart, lung, kidney, etc. (3–6). However, as “the window to the soul”, the eye has attracted relatively less attention in the field of rheumatology.

Glaucoma is a heterogeneous group of ocular diseases featured by degeneration of the optic nerve and retinal ganglion cell loss with multifactorial pathogenic causes (7). Currently, glaucoma is the leading global cause of irreversible blindness, with an estimated prevalence of 76.0 million cases worldwide (8). In 2020 alone, glaucoma has caused 3.6 million cases of blindness (9). Traditionally, increased eye pressure (intraocular pressure) has been viewed as a major risk factor for glaucoma development and progression (10). However, increased intraocular pressure is seen in approximately 70% of patients only, and some patients still exhibit disease progression despite the intraocular pressure being controlled within normal ranges, both of which indicate that there are other risk factors for glaucoma (11, 12). Taken together, it is crucial to explore the unidentified risk factors for glaucoma, which will facilitate early detection and timely treatment so as to reduce glaucoma-related blindness.

Notably, both autoimmunity and inflammation, the main characteristics of rheumatic diseases, have been found to be related to glaucoma (11, 13–15). This inspired us to speculate whether there is an association between rheumatic diseases and glaucoma.

Mendelian randomization (MR) is an established approach for making causal inferences in epidemiology (16). MR uses genetic variants identified in the genome-wide association studies (GWAS) as instrumental variables (IVs) (17). In MR, these IVs are used to assess the causal effects of defined exposures on a phenotype. At birth, according to Mendel’s second law, an individual is naturally assigned to either carry some IVs or not (18). Those who have inherited such IVs are steadily affected by IV-related exposures. When these individuals grow up, they either show a phenotype of interest (e.g., a disease) or not. Since these IVs are fixed at conception and typically not subjected to confounders, the differences in the studied phenotype are attributed to the differences in the exposure (18). For instance, if some IVs are strongly associated with depression (the exposure) and also associated with a higher risk of breast cancer (the phenotype), then it is likely that depression is causally associated with the risk of breast cancer (19).

In this study, we included six common rheumatic diseases, namely, ankylosing spondylitis (AS), rheumatoid arthritis (RA), systemic lupus erythematosus (SLE), Sicca syndrome/Sjögren’s syndrome (SS), dermatomyositis (DM), and gout. Glaucoma was divided into two categories: primary open-angle glaucoma (POAG) and primary angle-closure glaucoma (PACG). We first conducted a two-sample MR to evaluate the potential causality between the six rheumatic diseases and the two types of glaucoma. Then, the results were further verified by multivariable MR (MVMR) to provide a robust conclusion.

## 2 Materials and methods

**Figure 1:**
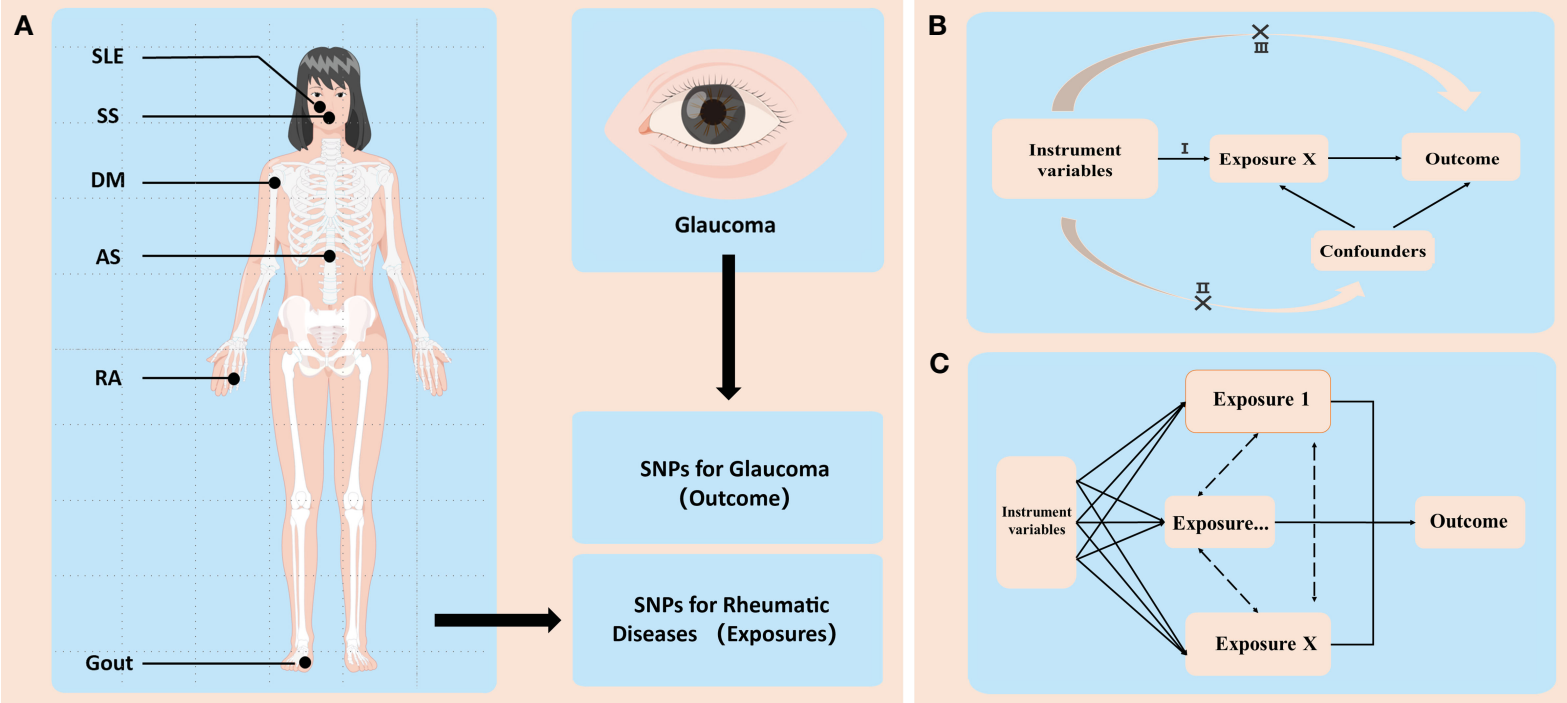
An overview of the study design. **(A)** GWAS (genome-wide association study) identified sequence variations, namely, single-nucleotide polymorphisms (SNPs), that exist throughout the human genome, and then screened disease-related SNPs from them. In this study, SNPs related with six common rheumatic diseases and two types of glaucoma were used. These rheumatic diseases included ankylosing spondylitis (AS), rheumatoid arthritis (RA), systemic lupus erythematosus (SLE), Sicca syndrome (SS), dermatomyositis (DM), and gout. Glaucoma is divided into two categories: primary open-angle glaucoma (POAG) and primary angle-closure glaucoma (PACG). **(B)** Illustration of Mendelian randomization (MR). MR has three main assumptions: (I) the instrumental variables (IVs) are associated with the exposure of interest (i.e., each rheumatic disease in this study); (II) the IVs are independent of potential confounders; and (III) the IVs influence the outcome (i.e., the two types of glaucoma) only through the exposure. **(C)** Illustration of multivariable MR (MVMR). In some cases, the IVs may be related to several interactive exposures (confounders) that may influence the same outcome. MVMR can be used to adjust for these confounders (e.g., diabetes, coronary heart disease, and smoking, etc.) and then analyze whether the exposure of interest has a direct impact on the outcome.

An overview of the study design is shown in **Figure 1**. No additional ethical approval is needed due to the use of the publicly available GWAS and FinnGen data.

### 2.1 Data sources

Data for AS, RA, and SLE were extracted from the published IEU Open GWAS database (https://gwas.mrcieu.ac.uk/). Data for SS, DM, and Gout were extracted from the FinnGen8 database (http://www.finngen.fi). The sample sizes for each rheumatic diseases were as follows: AS (9,069 cases and 1,550 controls), RA (14,361 cases and 43,923 controls), SLE (5,201 cases and 9,066 controls), SS (2,247 cases and 332,115 controls), DM (363 cases and 261,098 controls), and gout (3,768 cases and 336,797 controls). Data for glaucoma obtained from the FinnGen8 database include POAG (6,585 cases and 326,434 controls) and PACG (1,062 cases and 326,434 controls). All individuals included in the analysis were of European ancestry, and the overlap between the cohort of rheumatic diseases and the cohort of glaucoma was less than 20%.

### 2.2 Selection of IVs

To explore the causal association between the six rheumatic diseases and two types of glaucoma, we first screened IVs for these diseases. Only single-nucleotide polymorphisms (SNPs) according to the following criteria were selected as IVs (1): SNPs had a genome-wide *p*-value < 5×10^−8^ so that the SNPs were strongly associated with the exposures (2); SNPs should have linkage disequilibrium (LD) *r* ^2^ < 0.001 and < 1,000 KB from the index variant (3); SNPs should have *F*-value ≥ 10, suggesting little possibility of weak IV bias. The formula is as follows: *F* = (β_exposure_/SE_exposure_)^2^, where β_exposure_ and SE_exposure_ were the effect value and standard error of the exposure, respectively. Moreover, outcome-related SNPs with a *p*-value > 1×10^−5^ were excluded. Then, we also checked the selected SNPs in Phenoscanner (www.phenoscanner.medschl.cam.ac.uk), a commonly used database for human genotype–phenotype associations, to test whether these SNPs were related to potential confounding factors (diabetes, hypertension, and glucocorticoid use) (20). Lastly, palindromic SNPs with intermediate allele frequencies were removed from the MR analyses.

### 2.3 MR analyses

First, a two-sample MR was used to detect the potential causal association between the six rheumatic diseases and the two types of glaucoma. Then, to provide a robust conclusion, we performed an MVMR to verify the results of two-sample MR by adjusting for type 2 diabetes, major coronary heart disease event, cigarette smoking, and systolic blood pressure.

We applied three different approaches to estimate the causal effects between the six rheumatic diseases and glaucoma, including MR Egger, weighted median, and the inverse-variance weighted (IVW) method. The IVW approach was used as our main analysis approach, with the other two used as auxiliary references. The three methods were based on three different assumptions. The IVW method assumes that there is no horizontal pleiotropy, either because all the SNPs used are valid IVs, or because the total pleiotropy is balanced (21). The MR-Egger method allows for horizontal pleiotropic effects under the premise that the SNPs meet the Instrument Strength Independent of Direct Effect (InSIDE) assumption (i.e., the SNP–exposure association is independent from the SNP–outcome association) (22). The slope of the MR-Egger regression provides the estimate of the causal association between the six rheumatic diseases and the two types of glaucoma. Moreover, MR-Egger also serves as a test of directional pleiotropy, namely, the MR-Egger intercept test (23). Moreover, the weighted median method can be performed to give an estimate for the causal effect when no more than half of the SNPs violate horizontal pleiotropy (24).

Several different methods for sensitivity analysis were performed to justify the MR results. The MR-Egger intercept test, as mentioned previously, was used to detect directional pleiotropy. Additionally, MR pleiotropy residual sum and outlier (MR-PRESSO) test were used to detect outlier SNPs, which would then be discarded. Furthermore, Cochran’s *Q* statistic was used to evaluate heterogeneity. Finally, leave-one-out analysis was performed by eliminating each SNP one by one to test if the results were driven by any single SNP.

### 2.4 Statistical analysis

All MR analyses were accomplished using the “TwoSampleMR” and “MRPRESSO” packages in R (version 4.2.3). The results were expressed as odds ratios (ORs) with 95% confidence intervals (95% CI) to quantify the magnitude of the causality between the six rheumatic diseases and the two types of glaucoma. The statistical significances were set as *p*-value < 0.05 when determining the causal effects between rheumatic diseases and glaucoma.

## 3 Results

### 3.1 IVs for rheumatic diseases and glaucoma

A total of 166 SNPs were selected to genetically predict rheumatic diseases, including 29 SNPs for AS, 82 SNPs for RA, 29 SNPs for SLE, 14 SNPs for SS, 5 SNPs for DM, and 7 SNPs for gout. Detailed information about these rheumatic disease-related SNPs are listed in **Supplementary Table 1**. F statistics for all SNPs were higher than 10 in this study, indicating a small chance of weak IV bias. In addition, the relationship between these SNPs and outcomes was weak, with all *p*-values> 1×10^−5^.

### 3.2 Two-sample MR between rheumatic diseases and glaucoma

**Figure 2:**
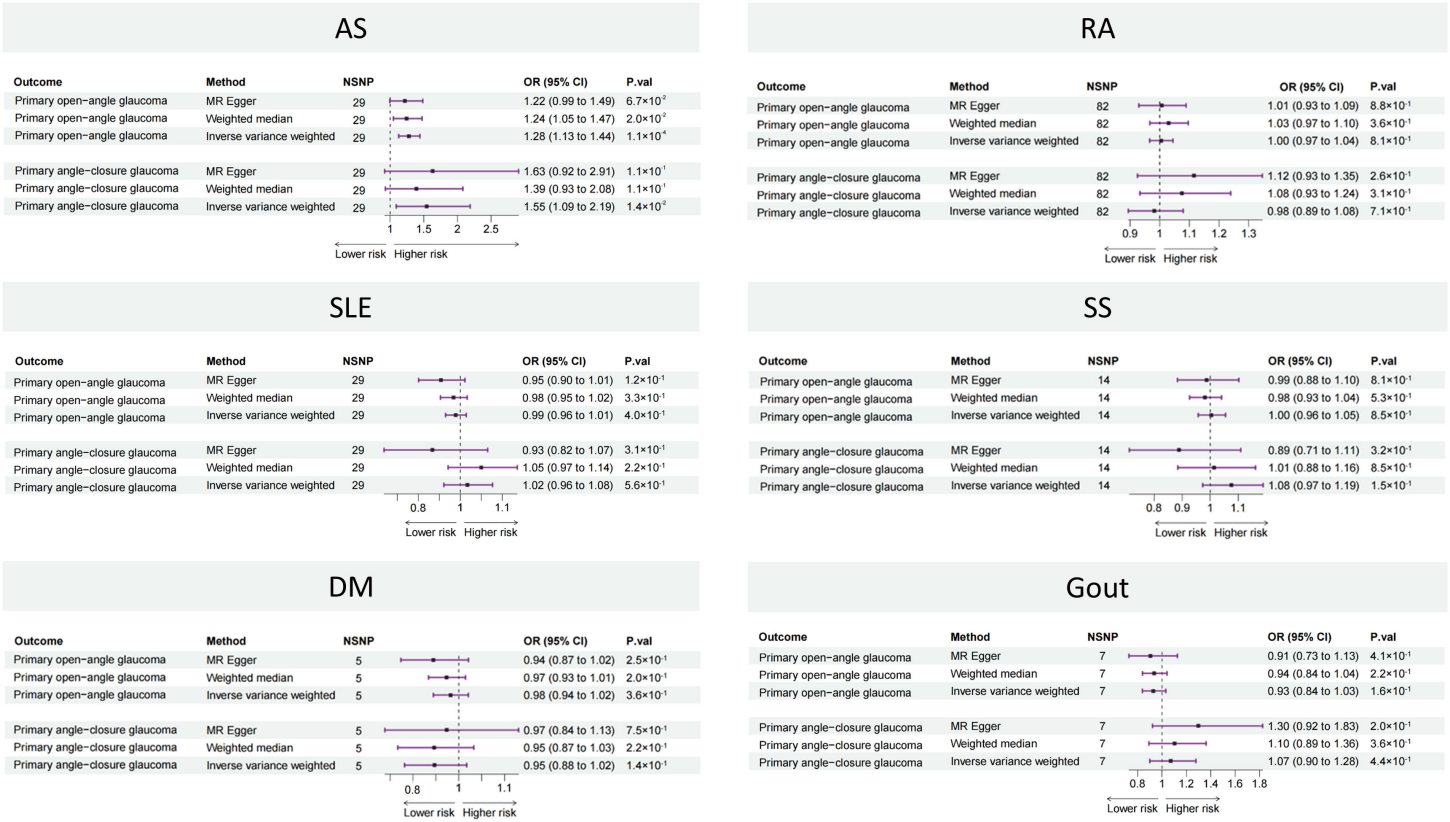
Forest plot showing causal estimates between rheumatic diseases and glaucoma in two-sample MR. In the forest plot, the dashed vertical line represents the ineffective line (OR = 1), the horizontal coordinate corresponding to each square represents the OR value calculated by different methods, and each horizontal solid line represents the 95% CI of the corresponding OR value. MR, Mendelian randomization; NSNP, number of single-nucleotide polymorphisms; OR, odds ratio; CI, confidence interval; AS, ankylosing spondylitis; RA, rheumatoid arthritis; SLE, systemic lupus erythematosus, SS, Sicca syndrome, DM, dermatomyositis.

The two-sample MR results between the six rheumatic diseases and two types of glaucoma are listed in **Figure 2**. The causal estimates from the IVW method showed that genetically predicted AS was positively associated with the risk of both POAG (OR: 1.28, 95% CI: 1.13 to 1.44, *p* = 1.1 × 10^−4^) and PACG (OR: 1.55, 95% CI: 1.09 to 2.09, *p* = 1.4 × 10^−2^). The other five rheumatic diseases (RA, SLE, SS, DM, and gout) showed no significant association with POAG or PACG.

The results of the Cochran’s *Q* statistic, MR-Egger intercept test, and MR-PRESSO test are listed in **Supplementary Table 2**. All Cochran’s *Q*-derived *p*-value s were > 0.05 except for estimates of SLE on POAG. All *p*-value s were > 0.05 in the MR-Egger intercept test, indicating that no horizontal pleiotropy existed. All *p*-value s were > 0.05 in the MR-PRESSO test, except for estimates of SLE on POAG. The leave-one-out analysis showed that the association between AS and glaucoma was not driven by any single SNP (**Supplementary Figure 1**).

The results of the sensitivity analyses indicated that the causal association between AS and glaucoma was robust.

### 3.3 Multivariable MR between rheumatic diseases and glaucoma

**Figure 3:**
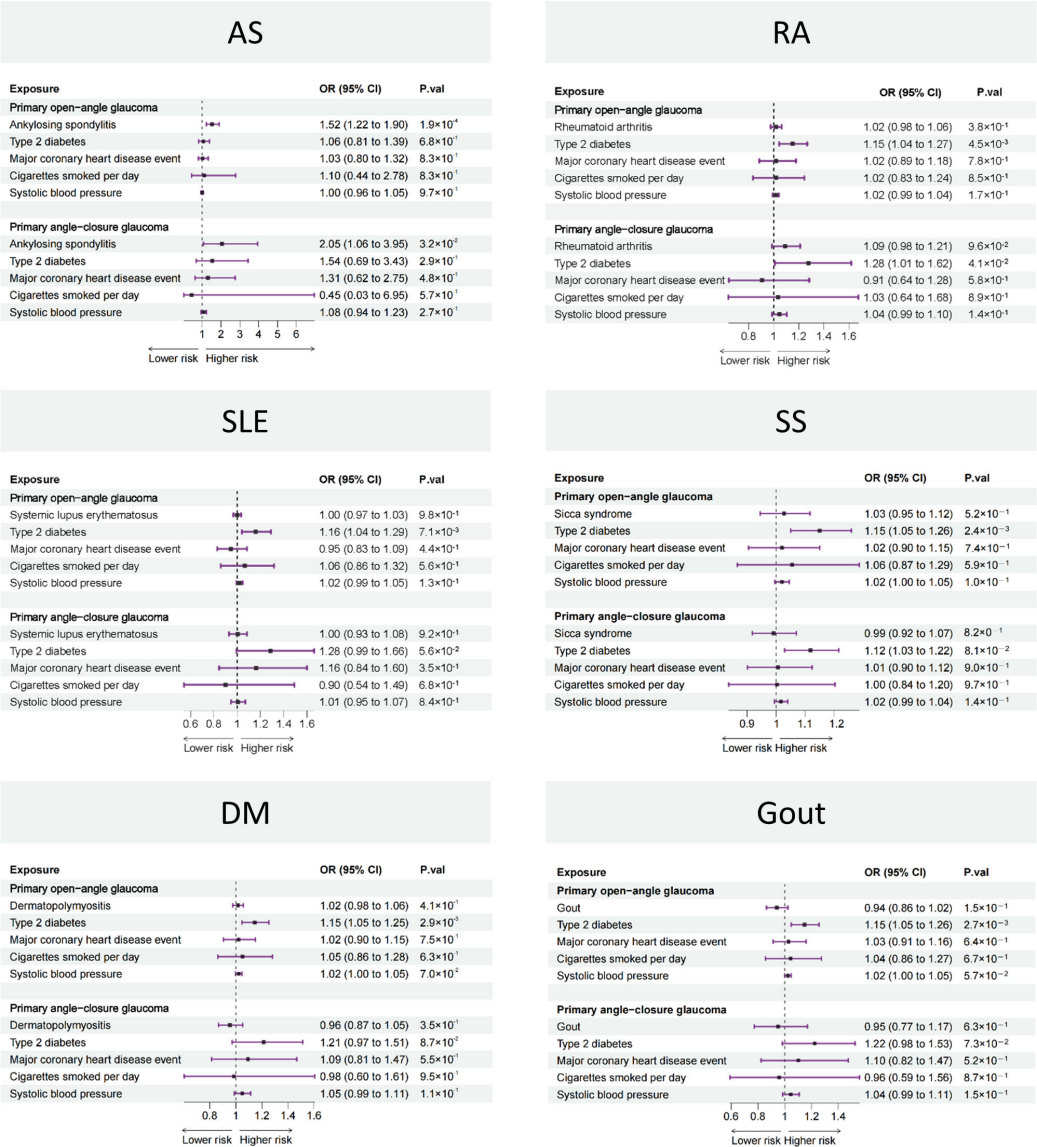
Forest plot showing causal estimates between rheumatic diseases and glaucoma in MVMR. In the forest plot, the dashed vertical line represents the ineffective line (OR = 1), the horizontal coordinate corresponding to each square represents the OR value calculated by different methods, and each horizontal solid line represents the 95% CI of the corresponding OR value. MVMR, multivariable Mendelian randomization; OR, odds ratio; CI, confidence interval; AS, ankylosing spondylitis; RA, rheumatoid arthritis; SLE, systemic lupus erythematosus, SS, Sicca syndrome, DM, dermatomyositis.

On the basis of the results of the two-sample MR, only AS was associated with the risk of POAG and PACG. Then, we further adjusted for type 2 diabetes, major coronary heart disease event, cigarette smoking, and systolic blood pressure in an MVMR to verify the reliability of the two-sample MR results (**Figure 3**). The MVMR results were in consistent with two-sample MR, i.e., AS was a risk factor for both POAG (OR: 1.52, 95% CI: 1.22 to 1.90, *p* = 1.9 × 10^−4^) and PACG (OR: 2.05, 95% CI: 1.06 to 3.95, *p* = 3.2 × 10^−2^) while the other five rheumatic diseases were not.

## 4 Discussion

This study, as far as we know, is the first to evaluate the causal association between the six common rheumatic diseases (AS, RA, SLE, SS, DM, and gout) and glaucoma. Using two-sample MR, we found that AS is associated with a higher risk of both POAG and PACG. Then, this finding was verified by MVMR, where several major confounders were adjusted for. In other words, AS is a risk factor for glaucoma, whereas RA, SLE, SS, DM, and gout were not.

It is well known that rheumatic diseases are a complex spectrum of diseases that affects multiple organs and systems (1). In fact, ocular involvement is a common extra-articular manifestation of several rheumatic diseases, including AS (25–28). For example, anterior uveitis is one of the most commonly seen extra-articular manifestations of AS, affecting 11.4%–15.8% of AS patients, and the prevalence of anterior uveitis can increase with a prolonged duration of AS (29–31). However, there are no published data about the prevalence of glaucoma in AS, which suggests that more attention needs to be given to this disease with irreversible vision loss in the AS group.

Owing to the limited relevant studies at present, the specific pathogenesis of glaucoma in AS is still unclear but could be contributed by three cornea-related mechanisms: central corneal thickness (CCT), corneal hysteresis (CH), and corneal stiffness. First, studies have revealed that AS patients have lower CCT when compared to healthy individuals and the CCT decreased with an increase in Bath Ankylosing Spondylitis Metrology Index score (BASMI) (32, 33). It is widely accepted that lower CCT brings a higher risk of glaucoma (34). Second, AS patients tend to have lower CH, and CH is negatively associated with the duration of disease (32, 33). Lower CH has been proven to be a risk factor for predicting both the development and progression of glaucoma (35–37). Third, it has been found that AS patients have lower corneal stiffness when compared with healthy individuals, as demonstrated by the lower stiffness parameter at first applanation (SPA1) (38). The abnormality of corneal biomechanics (including corneal stiffness) is considered to be an important risk factor for the development and progression of normal-tension glaucoma (39, 40).

AS is an inflammatory collagen connective tissue disease. The cornea, because of its high collagen content, is a target tissue of AS (41). The corneal stroma is the main part of the cornea, accounting for approximately 90% of the total corneal thickness, and is composed of collagen fibrils that are arranged in parallel into lamellae (32, 42). The lamellar organization of the collagen fibrils plays an important role in the biomechanical properties of the human cornea, such as maintaining its shape and strength (38). Thus, it is possible that collagen alterations caused by the inflammatory pathological processes in AS could affect the biomechanical parameters of the cornea, leading to a higher risk of glaucoma (38, 43).

Glaucoma is currently the leading cause of irreversible vision loss all over the world (8). Despite considerable progress in its treatment over the past few decades, the optic nerve damage and retinal ganglion cell loss caused by glaucoma are still irreversible (44). Nevertheless, diagnosis of glaucoma is often delayed since patients could remain asymptomatic until a relatively advanced stage (44). Additionally, the decrease in vision-related quality of life can be present as early as before patients are unaware of their having glaucoma, highlighting the irreplaceable role of early diagnosis and treatment (45). To sum up, recognizing the risk factors of glaucoma may aid in targeted screening among high-risk populations (e.g., patients with AS) and finally to help prevent or delay blindness in these patients. Considering the high incidence of AS in the general population (0.1%–0.5%), it is necessary to further explore the relationship between AS and glaucoma in future studies (46).

There are several strengths worth noting in this study. Above all, this is the first study to evaluate the causal association between the six common rheumatic diseases and glaucoma. Moreover, traditional observational studies are affected by reverse causality and confounding factors and, therefore, are less reliable when making causal inferences. The MR design used in this study can avoid reverse causality and most confounding factors. Moreover, an MR study is more convenient, cost-effective, and labor-saving when compared with traditional studies.

Some limitations need to be acknowledged in this study. First, although we have used the MR-PRESSO test to screen and discard outlier SNPs, the potential effect of heterogeneity on the study results cannot be completely ruled out. Second, since the enrolled participants were mainly of European ancestry, our results may not necessarily be generalizable to populations of other races. Third, although our study has revealed the causal association between AS and glaucoma, the exact pathophysiological mechanisms are still unclear, and further studies are needed. Last, it should be noticed that the causal association between AS and glaucoma might be partially mediated by some intermediate phenotypes, such as anterior uveitis. However, this does not change the finding that AS is a risk factor for glaucoma.

## 5 Conclusions

This is the first MR study to investigate the causal association between six common rheumatic diseases (AS, RA, SLE, SS, DM, and gout) and two types of glaucoma (POAG and PACG). We have found a causal association between AS and both types of glaucoma (i.e., AS is a risk factor for both POAG and PACG). We stress the importance of glaucoma screening for AS patients, which would help in early diagnosis and prevention of irreversible vision loss. We urge future studies to further explore the underlying mechanisms.

## Data availability statement

The original contributions presented in the study are included in the article/**Supplementary Material**. Further inquiries can be directed to the corresponding authors.

## Ethics statement

Ethical approval was not required for the study involving humans in accordance with the local legislation and institutional requirements. Written informed consent to participate in this study was not required from the participants or the participants’ legal guardians/next of kin in accordance with the national legislation and the institutional requirements.

## Author contributions

YM, ZT, LL, and CC designed the study. YM, ZT, and YS analyzed and interpreted the data. YM and ZT were major contributors in writing the manuscript. LL and CC reviewed and edited the manuscript. All authors contributed to the article and approved the submitted version.
