# Supplementary material for: Causal association between common rheumatic diseases and glaucoma: a Mendelian randomization study
Source: Front Immunol. 2023 Sep 19;14:1227138. doi: 10.3389/fimmu.2023.1227138 (PMC10550209; doi:10.3389/fimmu.2023.1227138)
Supplement: Supplementary file 1 [file DataSheet_1.pdf]

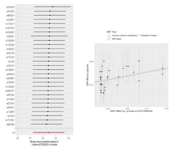

AS to POAG

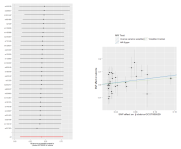

AS to PACG

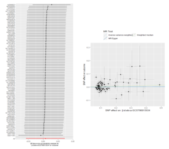

RA to POAG

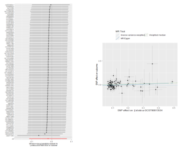

RA to POAG

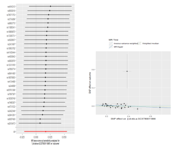

SLE to POAG

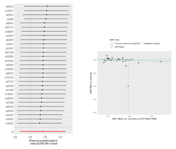

SLE to POAG

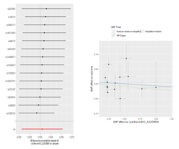

SS to POAG

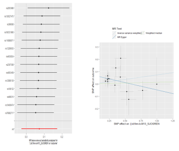

SS to POAG

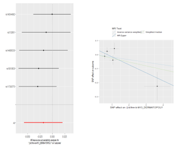

DM to POAG

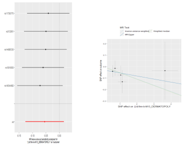

DM to POAG

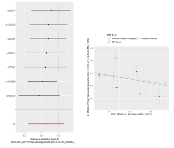

Gout to POAG

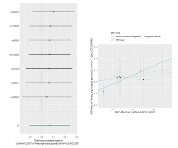

Gout to POAG
