# Supplementary material for: Causal association between common rheumatic diseases and glaucoma: a Mendelian randomization study
Source: Front Immunol. 2023 Sep 19;14:1227138. doi: 10.3389/fimmu.2023.1227138 (PMC10550209; doi:10.3389/fimmu.2023.1227138)
Supplement: Supplementary file 2 [file Table_1.docx]

***Supplementary Material***

**Genetic Causal Association between Common Rheumatic Diseases and Glaucoma: A Mendelian Randomization Study**

**Yang Meng^1†^, Zongbiao Tan^2†^, Yu Su ^1^, Lu Li ^1*^ and Changzheng Chen ^1*^**

*** Correspondence:** Lu Li (lilu-000000@163.com), Changzheng Chen ([whuchenchzh@163.com](mailto:whuchenchzh@163.com))

**Supplementary Table 1**

**SNPs for the six rheumatic diseases**

| **SNP** | **Chr** | **Pos** | **Effect allele** | **Other allele** | **Association with Exposures** | | | | **Association with POAG** | | | **Association with PACG** | | |
| --- | --- | --- | --- | --- | --- | --- | --- | --- | --- | --- | --- | --- | --- | --- |
|  |  |  |  |  | **Se** | **Beta** | **Pval** | **F** | **Beta** | **Se** | **Pval** | **Beta** | **Se** | **Pval** |
| **Instrumental Variables of SLE** | | | | | | | | | | | | | | |
| rs10036748 | 5 | 150458146 | T | C | 0.0338 | 0.2021 | 2.29E-09 | 36 | 0.020037 | 0.020092 | 0.318639 | 0.008553 | 0.048957 | 0.861308 |
| rs10953792 | 7 | 75172937 | C | T | 0.032 | 0.1994 | 4.69E-10 | 39 | -0.00427 | 0.0209 | 0.838283 | 0.050863 | 0.050886 | 0.317535 |
| rs11889341 | 2 | 191943742 | T | C | 0.0295 | 0.4164 | 3.33E-45 | 199 | -0.00865 | 0.02131 | 0.684668 | 0.036669 | 0.051906 | 0.479907 |
| rs13213165 | 6 | 33039729 | G | T | 0.0322 | 0.2888 | 3.13E-19 | 80 | 0.001587 | 0.028871 | 0.956164 | 0.028348 | 0.070575 | 0.687926 |
| rs13385731 | 2 | 33701890 | C | T | 0.0421 | -0.3654 | 4.19E-18 | 75 | 0.026375 | 0.042224 | 0.532206 | 0.228134 | 0.103495 | 0.027503 |
| rs16869875 | 6 | 32192217 | T | C | 0.04 | 0.4811 | 3.01E-33 | 145 | -0.06387 | 0.061856 | 0.301832 | -0.1046 | 0.150744 | 0.487741 |
| rs16870693 | 6 | 32711691 | A | C | 0.0403 | 0.3849 | 1.37E-21 | 91 | 0.758166 | 0.37324 | 0.0422231 | -1.00096 | 0.981298 | 0.307713 |
| rs2422349 | 1 | 173340809 | T | G | 0.0554 | 0.3307 | 2.35E-09 | 36 | 0.033648 | 0.022488 | 0.134583 | 0.02451 | 0.054557 | 0.653245 |
| rs2431697 | 5 | 159879978 | C | T | 0.0451 | -0.2538 | 1.84E-08 | 32 | 0.003613 | 0.01847 | 0.844919 | -0.00569 | 0.044847 | 0.899104 |
| rs244689 | 5 | 133422816 | G | A | 0.0291 | -0.1595 | 4.29E-08 | 30 | -0.01144 | 0.020692 | 0.580385 | 0.095638 | 0.050257 | 0.0570453 |
| rs2618473 | 8 | 11344127 | T | C | 0.0339 | 0.359 | 2.89E-26 | 112 | 0.052674 | 0.020333 | 0.009583 | -0.03403 | 0.049833 | 0.494745 |
| rs2841281 | 14 | 105394669 | T | C | 0.0292 | 0.1933 | 3.56E-11 | 44 | 0.044008 | 0.018314 | 0.0162596 | 0.074344 | 0.04445 | 0.094417 |
| rs3734266 | 6 | 34823187 | C | T | 0.0399 | 0.334 | 6.09E-17 | 70 | -0.05383 | 0.033257 | 0.105501 | 0.087723 | 0.080903 | 0.278234 |
| rs4134466 | 6 | 106577368 | G | A | 0.0306 | -0.2136 | 3.09E-12 | 49 | -0.00695 | 0.018149 | 0.70182 | -0.02393 | 0.044169 | 0.588002 |
| rs41430444 | 2 | 191878487 | C | T | 0.0428 | -0.3077 | 6.57E-13 | 52 | 0.003196 | 0.032068 | 0.920618 | -0.01085 | 0.078353 | 0.889839 |
| rs4731532 | 7 | 128572766 | A | G | 0.0334 | 0.3716 | 8.15E-29 | 124 | 0.017846 | 0.017982 | 0.320987 | 0.021273 | 0.043651 | 0.626025 |
| rs4916315 | 1 | 173215372 | T | C | 0.0311 | 0.3358 | 4.12E-27 | 117 | -0.03845 | 0.020655 | 0.0626888 | -0.05215 | 0.050171 | 0.298562 |
| rs4930642 | 11 | 68816370 | G | A | 0.0344 | -0.2336 | 1.10E-11 | 46 | 0.033532 | 0.061026 | 0.582688 | -0.00676 | 0.148253 | 0.963652 |
| rs5029937 | 6 | 138195151 | T | G | 0.0676 | 0.6844 | 4.32E-24 | 103 | -0.02887 | 0.067805 | 0.670252 | -0.05525 | 0.166651 | 0.740234 |
| rs55701306 | 17 | 16842447 | T | C | 0.0293 | 0.1662 | 1.38E-08 | 32 | 0.040699 | 0.036179 | 0.260623 | -0.02092 | 0.088256 | 0.812584 |
| rs6941485 | 6 | 250619 | G | A | 0.0314 | 0.2034 | 9.53E-11 | 42 | 0.004475 | 0.02043 | 0.826603 | 0.070249 | 0.04962 | 0.15685 |
| rs6993775 | 8 | 11369989 | T | G | 0.0346 | 0.2987 | 6.06E-18 | 75 | -0.01977 | 0.020907 | 0.34427 | 0.015987 | 0.05042 | 0.751185 |
| rs7097397 | 10 | 50025396 | A | G | 0.031 | -0.2385 | 1.32E-14 | 59 | -0.01481 | 0.018633 | 0.426857 | -0.08003 | 0.045219 | 0.0767574 |
| rs7486387 | 12 | 12866911 | A | G | 0.0314 | -0.1886 | 1.98E-09 | 36 | -0.04929 | 0.026522 | 0.063103 | -0.15983 | 0.06436 | 0.0130167 |
| rs7650774 | 3 | 119205050 | C | T | 0.0306 | -0.1798 | 4.07E-09 | 35 | 0.001628 | 0.02708 | 0.952073 | 0.025471 | 0.066092 | 0.69995 |
| rs76571753 | 7 | 73974915 | T | G | 0.0414 | 0.2792 | 1.46E-11 | 45 | -0.03954 | 0.023599 | 0.0938512 | -0.02629 | 0.057209 | 0.645865 |
| rs9262670 | 6 | 31034990 | T | C | 0.0324 | -0.2252 | 3.51E-12 | 48 | 1.38E-05 | 0.020011 | 0.999449 | 0.009771 | 0.048687 | 0.840938 |
| rs9387400 | 6 | 116694120 | A | C | 0.0536 | -0.2967 | 3.14E-08 | 31 | -0.04824 | 0.018056 | 0.0075445 | -0.04638 | 0.0439 | 0.290796 |
| rs9938016 | 16 | 86002524 | T | C | 0.0591 | -0.3974 | 1.78E-11 | 45 | 0.023417 | 0.020262 | 0.247783 | 0.002279 | 0.048987 | 0.962901 |
| **Instrumental Variables of SS** | | | | | | | | | | | | | | |
| rs10409474 | 19 | 850733 | G | C | 0.0674 | 0.3521 | 1.78E-07 | 27 | 0.002409 | 0.029807 | 0.935584 | 0.129346 | 0.072405 | 0.0740321 |
| rs116898071 | 11 | 58601814 | G | A | 0.2124 | 0.9988 | 2.57E-06 | 22 | -0.01247 | 0.087517 | 0.886661 | -0.00098 | 0.214926 | 0.996368 |
| rs12888138 | 18 | 53822656 | T | C | 0.0519 | -0.2463 | 2.09E-06 | 23 | -0.06564 | 0.023459 | 0.0051427 | -0.04284 | 0.056208 | 0.445917 |
| rs13289503 | 9 | 110186251 | C | T | 0.0441 | -0.2243 | 3.67E-07 | 26 | -0.00599 | 0.01972 | 0.761178 | -0.01966 | 0.048039 | 0.682427 |
| rs138527475 | 4 | 6942816 | G | C | 0.1212 | 0.5556 | 4.57E-06 | 21 | 0.024717 | 0.052558 | 0.638147 | -0.12503 | 0.129135 | 0.332948 |
| rs190331960 | 6 | 27843694 | A | G | 0.0737 | 0.3387 | 4.37E-06 | 21 | -0.01852 | 0.025161 | 0.4616 | -0.00963 | 0.060736 | 0.873982 |
| rs199132 | 6 | 23503752 | A | C | 0.0449 | 0.2308 | 2.73E-07 | 26 | -0.02886 | 0.019975 | 0.148582 | 0.04424 | 0.048522 | 0.361896 |
| rs2307308 | 6 | 24784436 | T | C | 0.0911 | 0.4228 | 3.48E-06 | 22 | 0.022597 | 0.037966 | 0.55172 | 0.05633 | 0.092986 | 0.544657 |
| rs2853986 | 6 | 31338844 | C | T | 0.0742 | 0.7754 | 1.43E-25 | 109 | -0.01682 | 0.031255 | 0.590526 | -0.05271 | 0.07641 | 0.490294 |
| rs396568 | 19 | 3135644 | A | G | 0.1083 | -0.5257 | 1.20E-06 | 24 | -0.08532 | 0.047712 | 0.0737531 | 0.06225 | 0.11325 | 0.582547 |
| rs4630834 | 20 | 23740255 | T | C | 0.0408 | 0.1909 | 2.97E-06 | 22 | 0.020854 | 0.018252 | 0.253233 | 0.023765 | 0.044355 | 0.592109 |
| rs4853459 | 2 | 191962096 | C | T | 0.0479 | -0.2215 | 3.70E-06 | 21 | 0.008765 | 0.02131 | 0.680837 | -0.03415 | 0.051907 | 0.510647 |
| rs496315 | 20 | 10047038 | C | T | 0.0408 | 0.2254 | 3.21E-08 | 31 | -0.01204 | 0.018141 | 0.506826 | 0.065693 | 0.044169 | 0.13693 |
| rs76882717 | 10 | 14768307 | T | C | 0.0933 | 0.43 | 4.05E-06 | 21 | -0.04984 | 0.041233 | 0.226733 | 0.207718 | 0.099793 | 0.0373895 |
| **Instrumental Variables of DM** | | | | | | | | | | | | | | |
| rs10010630 | 4 | 8655375 | C | T | 0.1537 | 0.7094 | 3.94E-06 | 21 | 0.023422 | 0.026796 | 0.382067 | -0.01542 | 0.065332 | 0.813432 |
| rs117393775 | 7 | 78965822 | T | G | 0.2451 | 1.1322 | 3.85E-06 | 21 | 0.042121 | 0.041052 | 0.304879 | -0.13286 | 0.099472 | 0.181667 |
| rs144895330 | 7 | 425214 | G | A | 0.1187 | 0.5641 | 2.03E-06 | 23 | -0.01152 | 0.020919 | 0.581716 | -0.04121 | 0.050862 | 0.417852 |
| rs149344982 | 1 | 21889760 | A | G | 0.7648 | 3.6492 | 1.83E-06 | 23 | -0.22587 | 0.101318 | 0.0257935 | -0.03632 | 0.251115 | 0.88499 |
| rs3132651 | 6 | 30287777 | G | A | 0.195 | 1.0409 | 9.40E-08 | 28 | -0.03939 | 0.03274 | 0.228947 | -0.07381 | 0.080281 | 0.357855 |
| **Instrumental Variables of AS** | | | | | | | | | | | | | | |
| rs11065898 | 12 | 111862575 | T | C | 0.004806 | 0.026252 | 4.71E-08 | 30 | -0.01629 | 0.022518 | 0.469312 | -0.09076 | 0.05488 | 0.0981861 |
| rs11190133 | 10 | 101278725 | T | C | 0.004494 | -0.03387 | 4.84E-14 | 57 | 0.006216 | 0.018398 | 0.735485 | -0.08705 | 0.04474 | 0.0516868 |
| rs11209026 | 1 | 67705958 | A | G | 0.009545 | -0.10358 | 1.94E-27 | 118 | -0.00848 | 0.043628 | 0.845821 | 0.072104 | 0.103853 | 0.4875 |
| rs1128905 | 9 | 139253839 | C | T | 0.004095 | -0.02372 | 6.95E-09 | 34 | -0.02806 | 0.017959 | 0.118151 | 0.004074 | 0.043638 | 0.925625 |
| rs11624293 | 14 | 88488821 | C | T | 0.006692 | 0.042868 | 1.49E-10 | 41 | 0.030542 | 0.032688 | 0.350118 | -0.00794 | 0.080168 | 0.921097 |
| rs1250550 | 10 | 81060317 | A | C | 0.004305 | -0.02604 | 1.46E-09 | 37 | -0.01621 | 0.018397 | 0.378292 | 0.007081 | 0.044639 | 0.873958 |
| rs12615545 | 2 | 182048452 | C | T | 0.004173 | 0.025473 | 1.03E-09 | 37 | -0.0122 | 0.017986 | 0.497431 | -0.10882 | 0.043718 | 0.0128027 |
| rs17312661 | 6 | 28300336 | G | A | 0.005729 | -0.10392 | 1.56E-73 | 329 | -0.05145 | 0.031152 | 0.0986211 | -0.07735 | 0.075949 | 0.308452 |
| rs1801274 | 1 | 161479745 | G | A | 0.004177 | 0.025318 | 1.35E-09 | 37 | 0.011617 | 0.017967 | 0.517905 | 0.079189 | 0.043635 | 0.0695521 |
| rs1860545 | 12 | 6446777 | A | G | 0.004354 | -0.02747 | 2.78E-10 | 40 | -0.00027 | 0.018173 | 0.988353 | -0.00898 | 0.044104 | 0.838601 |
| rs213205 | 6 | 33241135 | C | T | 0.004178 | -0.0282 | 1.49E-11 | 46 | -0.03995 | 0.017939 | 0.0259639 | -0.02185 | 0.04358 | 0.616111 |
| rs2517653 | 6 | 30121185 | A | G | 0.004497 | 0.092853 | 1.04E-94 | 426 | 0.026798 | 0.018882 | 0.15583 | 0.108048 | 0.045894 | 0.0185567 |
| rs2531875 | 17 | 26148167 | T | G | 0.004245 | -0.02732 | 1.22E-10 | 41 | -0.00163 | 0.018554 | 0.929892 | -0.09359 | 0.045159 | 0.0382252 |
| rs2596501 | 6 | 31321211 | T | C | 0.004194 | -0.15234 | 1.00E-200 | 1319 | -0.03492 | 0.018044 | 0.0529651 | -0.05093 | 0.043806 | 0.245002 |
| rs27529 | 5 | 96126308 | G | A | 0.004299 | -0.06204 | 3.28E-47 | 208 | -0.01677 | 0.018759 | 0.371441 | 0.013534 | 0.045678 | 0.767003 |
| rs2836883 | 21 | 40466744 | A | G | 0.004748 | -0.03968 | 6.46E-17 | 70 | -0.01785 | 0.020834 | 0.391509 | -0.05538 | 0.050797 | 0.275592 |
| rs34932751 | 6 | 25762241 | C | T | 0.012522 | 0.081764 | 6.59E-11 | 43 | 0.039558 | 0.041035 | 0.335032 | 0.064635 | 0.098725 | 0.512664 |
| rs35164067 | 19 | 10525181 | A | G | 0.00495 | -0.03108 | 3.43E-10 | 39 | 0.017787 | 0.023492 | 0.448968 | 0.055172 | 0.057468 | 0.33703 |
| rs3857546 | 6 | 26157762 | T | C | 0.006525 | -0.07818 | 4.44E-33 | 144 | -0.04017 | 0.03969 | 0.311477 | -0.1726 | 0.097874 | 0.0778252 |
| rs4129267 | 1 | 154426264 | T | C | 0.004226 | -0.03077 | 3.32E-13 | 53 | 0.017433 | 0.019634 | 0.374611 | -0.09251 | 0.047866 | 0.0532721 |
| rs41299637 | 1 | 200877850 | G | T | 0.00491 | -0.03905 | 1.81E-15 | 63 | -0.01479 | 0.021864 | 0.498726 | 0.014222 | 0.053262 | 0.789456 |
| rs4248166 | 6 | 32366421 | C | T | 0.004923 | 0.152206 | 1.00E-200 | 956 | 0.022995 | 0.018958 | 0.225136 | 0.045338 | 0.046028 | 0.324614 |
| rs4672505 | 2 | 62560332 | G | A | 0.004151 | -0.05978 | 5.14E-47 | 207 | 0.000726 | 0.019036 | 0.969583 | -0.0162 | 0.046225 | 0.725952 |
| rs4676410 | 2 | 241563739 | A | G | 0.004902 | 0.028101 | 9.90E-09 | 33 | 0.028277 | 0.020318 | 0.164009 | 0.009576 | 0.049335 | 0.846095 |
| rs6556416 | 5 | 158818745 | C | A | 0.0046 | 0.025215 | 4.22E-08 | 30 | 0.015487 | 0.020617 | 0.452562 | 0.004157 | 0.050447 | 0.934327 |
| rs6600247 | 1 | 25305114 | C | T | 0.004151 | 0.032833 | 2.58E-15 | 63 | -0.0122 | 0.018002 | 0.497883 | 0.046138 | 0.043811 | 0.292289 |
| rs7191548 | 16 | 28614734 | C | T | 0.004285 | 0.024985 | 5.51E-09 | 34 | 0.001268 | 0.018201 | 0.94447 | -0.02938 | 0.044219 | 0.506437 |
| rs7746199 | 6 | 27261324 | T | C | 0.0058 | -0.07182 | 3.24E-35 | 153 | -0.03464 | 0.034209 | 0.311245 | -0.13085 | 0.084134 | 0.11988 |
| rs9901869 | 17 | 45575206 | A | G | 0.004089 | 0.031904 | 6.04E-15 | 61 | 0.050613 | 0.017916 | 0.0047281 | -0.06719 | 0.043653 | 0.123763 |
| **Instrumental Variables of RA** | | | | | | | | | | | | | | |
| rs10435844 | 9 | 123668199 | T | G | 0.0121 | -0.0784 | 9.73E-11 | 42 | 0.017441 | 0.018085 | 0.334861 | 0.007528 | 0.044041 | 0.864275 |
| rs10484404 | 6 | 28055495 | T | C | 0.0202 | -0.2304 | 3.95E-30 | 130 | -0.09435 | 0.043824 | 0.0313278 | 0.010856 | 0.105737 | 0.918225 |
| rs10807036 | 6 | 28606397 | A | G | 0.0265 | 0.3394 | 1.64E-37 | 164 | 0.032052 | 0.098544 | 0.744988 | 0.012618 | 0.244031 | 0.958763 |
| rs10911902 | 1 | 186632317 | T | C | 0.0152 | -0.0847 | 2.36E-08 | 31 | -0.01333 | 0.021699 | 0.53898 | -0.00068 | 0.052842 | 0.989674 |
| rs11123811 | 2 | 100760172 | C | T | 0.0114 | -0.0995 | 2.01E-18 | 76 | -0.03224 | 0.01801 | 0.0734108 | 0.037005 | 0.043812 | 0.398315 |
| rs11265409 | 1 | 160430247 | T | C | 0.0321 | 0.2064 | 1.2E-10 | 41 | 0.038589 | 0.072804 | 0.596085 | 0.163084 | 0.175052 | 0.351525 |
| rs114556512 | 16 | 23888311 | A | G | 0.0174 | -0.0962 | 3.41E-08 | 31 | 0.010771 | 0.022105 | 0.626072 | 0.009719 | 0.053451 | 0.855712 |
| rs114744895 | 6 | 34592450 | C | A | 0.0512 | -0.2926 | 1.11E-08 | 33 | -0.06201 | 0.110817 | 0.575761 | 0.130376 | 0.262561 | 0.619502 |
| rs115513538 | 6 | 36035753 | A | G | 0.0621 | -0.3922 | 2.62E-10 | 40 | -0.0561 | 0.070487 | 0.426103 | 0.051269 | 0.171299 | 0.764714 |
| rs11574914 | 9 | 34710338 | A | G | 0.0149 | 0.1153 | 9.92E-15 | 60 | -0.00909 | 0.020042 | 0.650266 | -0.00826 | 0.048755 | 0.865546 |
| rs117026326 | 7 | 74126034 | T | C | 0.0424 | 0.381 | 2.45E-19 | 81 | -0.03403 | 0.042298 | 0.421036 | -0.10116 | 0.103636 | 0.329018 |
| rs11889341 | 2 | 191943742 | T | C | 0.0129 | 0.1466 | 4.32E-30 | 129 | -0.00865 | 0.02131 | 0.684668 | 0.036669 | 0.051906 | 0.479907 |
| rs12173764 | 6 | 31314012 | T | C | 0.0121 | 0.1115 | 2.18E-20 | 85 | 0.028199 | 0.018191 | 0.121107 | 0.065047 | 0.04424 | 0.141478 |
| rs1234313 | 1 | 173166247 | G | A | 0.0133 | 0.0797 | 1.9E-09 | 36 | 0.006989 | 0.019222 | 0.716166 | -0.04239 | 0.046777 | 0.364846 |
| rs12530098 | 6 | 14107197 | T | C | 0.0204 | 0.1382 | 1.35E-11 | 46 | -0.01321 | 0.032612 | 0.685487 | -0.02264 | 0.0792 | 0.775015 |
| rs12795702 | 11 | 128156314 | G | A | 0.0144 | -0.0845 | 4.09E-09 | 34 | 0.000696 | 0.018338 | 0.969707 | 0.024545 | 0.044558 | 0.581736 |
| rs12918327 | 16 | 30626616 | T | C | 0.0157 | 0.0867 | 3.04E-08 | 30 | -0.00791 | 0.021238 | 0.709555 | -0.01963 | 0.051825 | 0.70492 |
| rs13103285 | 4 | 10726520 | T | C | 0.0131 | 0.0989 | 4.29E-14 | 57 | -0.03485 | 0.017991 | 0.0527157 | -0.02863 | 0.043791 | 0.51325 |
| rs1355208 | 2 | 30445322 | G | A | 0.0119 | 0.0818 | 6.77E-12 | 47 | 0.009144 | 0.018232 | 0.615976 | -0.02012 | 0.044272 | 0.649521 |
| rs1538981 | 10 | 31411355 | T | C | 0.0114 | 0.0671 | 4.42E-09 | 35 | -0.0047 | 0.018291 | 0.797054 | -0.08711 | 0.044467 | 0.0501118 |
| rs1571878 | 6 | 167540842 | T | C | 0.0116 | -0.1539 | 4.13E-40 | 176 | 0.009853 | 0.018119 | 0.586561 | 0.034359 | 0.044071 | 0.435614 |
| rs1595260 | 14 | 69267695 | T | A | 0.0126 | 0.0845 | 2.29E-11 | 45 | -0.00272 | 0.01908 | 0.88645 | -0.01057 | 0.046466 | 0.820035 |
| rs1858037 | 2 | 65598300 | A | T | 0.0131 | -0.1012 | 1.14E-14 | 60 | 0.02832 | 0.019765 | 0.15191 | 0.071216 | 0.048102 | 0.138735 |
| rs1883832 | 20 | 44746982 | C | T | 0.0127 | 0.1052 | 1.13E-16 | 69 | 0.016211 | 0.020236 | 0.423086 | -0.0662 | 0.049202 | 0.178498 |
| rs1893592 | 21 | 43855067 | C | A | 0.0132 | -0.0976 | 1.48E-13 | 55 | 0.020533 | 0.019404 | 0.289973 | 0.079863 | 0.047024 | 0.0894417 |
| rs1950897 | 14 | 68760141 | T | C | 0.0144 | 0.1069 | 1.02E-13 | 55 | 0.010644 | 0.019452 | 0.584254 | -0.03704 | 0.047074 | 0.431345 |
| rs2069235 | 22 | 39747780 | A | G | 0.014 | 0.1296 | 1.69E-20 | 86 | -0.03426 | 0.019134 | 0.0733483 | 0.0477 | 0.04642 | 0.304149 |
| rs2076616 | 1 | 17412501 | G | A | 0.0135 | -0.0885 | 6.2E-11 | 43 | -0.00658 | 0.018597 | 0.723431 | 0.044362 | 0.045207 | 0.32644 |
| rs212389 | 6 | 159489791 | A | G | 0.0147 | 0.1058 | 6.66E-13 | 52 | -0.01228 | 0.019813 | 0.535367 | -0.09758 | 0.048316 | 0.043419 |
| rs2233424 | 6 | 44233921 | T | C | 0.0187 | 0.1964 | 6.49E-26 | 110 | 0.031737 | 0.036409 | 0.383386 | 0.089556 | 0.087895 | 0.308249 |
| rs2258734 | 1 | 2483961 | A | G | 0.0123 | -0.0921 | 6.04E-14 | 56 | -0.03694 | 0.018898 | 0.0506174 | -0.01245 | 0.045808 | 0.785742 |
| rs2275806 | 10 | 8095340 | A | G | 0.0122 | -0.0725 | 2.51E-09 | 35 | 0.012892 | 0.018087 | 0.475977 | 0.04401 | 0.044005 | 0.31725 |
| rs2301888 | 1 | 17672730 | A | G | 0.0121 | -0.1282 | 3.75E-26 | 112 | -0.00228 | 0.019136 | 0.905118 | 0.008796 | 0.04646 | 0.849833 |
| rs244685 | 5 | 133423890 | G | T | 0.0144 | -0.089 | 6.04E-10 | 38 | -0.01015 | 0.020665 | 0.623459 | 0.09655 | 0.050184 | 0.0543638 |
| rs28411352 | 1 | 38278579 | T | C | 0.0136 | 0.0914 | 1.66E-11 | 45 | -0.0156 | 0.019855 | 0.432163 | -0.01309 | 0.048085 | 0.785518 |
| rs28421442 | 2 | 74219615 | A | T | 0.0214 | -0.1234 | 7.86E-09 | 33 | -0.06145 | 0.093109 | 0.509249 | -0.11896 | 0.224056 | 0.595472 |
| rs2847297 | 18 | 12797694 | G | A | 0.0119 | 0.0903 | 2.65E-14 | 58 | -0.02317 | 0.018917 | 0.220702 | -0.03002 | 0.045968 | 0.513674 |
| rs2856822 | 6 | 33047432 | C | A | 0.0123 | -0.2375 | 1.25E-82 | 373 | 0.059453 | 0.020434 | 0.0036199 | 0.029963 | 0.050288 | 0.55129 |
| rs28749541 | 6 | 29947532 | C | A | 0.0214 | -0.2831 | 5.13E-40 | 175 | -0.03049 | 0.030093 | 0.310927 | 0.128008 | 0.073839 | 0.082987 |
| rs2918392 | 5 | 10704797 | C | T | 0.0122 | 0.0668 | 4.62E-08 | 30 | -0.00057 | 0.019785 | 0.976968 | -0.02057 | 0.048087 | 0.668772 |
| rs3134883 | 10 | 6100725 | A | G | 0.0125 | 0.0991 | 1.98E-15 | 63 | -0.00032 | 0.01868 | 0.986232 | 0.06041 | 0.045508 | 0.184355 |
| rs34046593 | 4 | 26111593 | A | G | 0.017 | 0.1422 | 7.17E-17 | 70 | -0.01893 | 0.019422 | 0.329633 | 0.031945 | 0.047081 | 0.497452 |
| rs34502849 | 11 | 72411787 | A | G | 0.014 | -0.0851 | 1.07E-09 | 37 | -0.00473 | 0.020284 | 0.815753 | 0.098252 | 0.049236 | 0.0459854 |
| rs34536443 | 19 | 10463118 | C | G | 0.0474 | -0.3801 | 1.08E-15 | 64 | 0.019461 | 0.01816 | 0.283878 | 0.016857 | 0.044167 | 0.702707 |
| rs3757387 | 7 | 128576086 | C | T | 0.0137 | 0.1236 | 1.87E-19 | 81 | 0.013833 | 0.018185 | 0.446833 | -0.0227 | 0.04415 | 0.607144 |
| rs3761959 | 1 | 157669278 | T | C | 0.0115 | 0.0744 | 9.64E-11 | 42 | -0.0118 | 0.018038 | 0.513088 | -0.10458 | 0.04378 | 0.016904 |
| rs3806624 | 3 | 27764623 | G | A | 0.0131 | 0.0863 | 3.94E-11 | 43 | 0.002339 | 0.021069 | 0.911611 | 0.03632 | 0.051395 | 0.479762 |
| rs42034 | 7 | 92239144 | G | A | 0.0153 | 0.0871 | 1.28E-08 | 32 | 0.025563 | 0.024146 | 0.289742 | 0.047844 | 0.058646 | 0.414613 |
| rs4409785 | 11 | 95311422 | C | T | 0.017 | 0.0982 | 7.85E-09 | 33 | 0.15562 | 0.121821 | 0.201445 | 0.026326 | 0.296688 | 0.929296 |
| rs4481423 | 6 | 25720761 | C | T | 0.0352 | 0.3051 | 4.11E-18 | 75 | -0.01656 | 0.018459 | 0.369516 | -0.05957 | 0.044958 | 0.185144 |
| rs4602367 | 3 | 17053499 | G | A | 0.0117 | 0.075 | 1.76E-10 | 41 | 0.036004 | 0.051007 | 0.480272 | 0.075511 | 0.12171 | 0.534985 |
| rs4713305 | 6 | 30252836 | T | A | 0.0129 | 0.2205 | 3.66E-65 | 292 | 0.031072 | 0.02373 | 0.190392 | 0.047932 | 0.057295 | 0.402828 |
| rs4717901 | 7 | 74016979 | C | A | 0.0349 | 0.249 | 9.52E-13 | 51 | -0.01137 | 0.024076 | 0.636818 | 0.08059 | 0.058566 | 0.168805 |
| rs4963581 | 12 | 24813281 | A | G | 0.0156 | 0.0856 | 3.75E-08 | 30 | 0.002378 | 0.019824 | 0.904537 | 0.015365 | 0.048158 | 0.749684 |
| rs502919 | 10 | 6517167 | C | T | 0.0134 | 0.0829 | 6.17E-10 | 38 | -0.02275 | 0.031698 | 0.473007 | 0.064678 | 0.076788 | 0.399624 |
| rs5754104 | 22 | 21916361 | A | G | 0.0139 | 0.0891 | 1.36E-10 | 41 | 0.04293 | 0.049621 | 0.386948 | -0.05305 | 0.120438 | 0.659605 |
| rs6011186 | 20 | 62484008 | T | C | 0.0171 | -0.1074 | 3.19E-10 | 39 | 0.087788 | 0.035573 | 0.013595 | -0.02065 | 0.087044 | 0.812508 |
| rs61410323 | 21 | 45678620 | G | A | 0.0171 | 0.0989 | 7.83E-09 | 33 | 0.013408 | 0.022674 | 0.554295 | -0.02561 | 0.05531 | 0.643364 |
| rs61828284 | 1 | 173299743 | T | C | 0.0348 | -0.2018 | 6.33E-09 | 34 | 0.032503 | 0.040263 | 0.419522 | -0.04831 | 0.097516 | 0.620289 |
| rs61850862 | 10 | 64041537 | T | C | 0.0287 | 0.1924 | 1.99E-11 | 45 | -0.02504 | 0.021885 | 0.252459 | 0.190776 | 0.050943 | 0.0001805 |
| rs62394491 | 6 | 25934106 | T | C | 0.0168 | 0.1059 | 2.77E-10 | 40 | 0.017663 | 0.021599 | 0.413492 | 0.079669 | 0.052534 | 0.129386 |
| rs62422878 | 6 | 106676470 | T | C | 0.0176 | 0.1037 | 3.57E-09 | 35 | -0.03444 | 0.020253 | 0.0890307 | 0.026836 | 0.049048 | 0.584279 |
| rs6589684 | 11 | 118610957 | A | G | 0.0153 | -0.1122 | 2.29E-13 | 54 | 0.074362 | 0.019631 | 0.0001518 | 0.066211 | 0.048176 | 0.169334 |
| rs660442 | 11 | 64042997 | A | G | 0.0175 | -0.1067 | 1.11E-09 | 37 | -0.01481 | 0.018633 | 0.426857 | -0.08003 | 0.045219 | 0.0767574 |
| rs6917489 | 6 | 36368169 | A | G | 0.0125 | 0.0683 | 4.64E-08 | 30 | 0.02409 | 0.023335 | 0.301896 | 0.072652 | 0.056509 | 0.198552 |
| rs7097397 | 10 | 50025396 | A | G | 0.012 | -0.0847 | 1.42E-12 | 50 | -0.01118 | 0.020476 | 0.585193 | 0.047179 | 0.049756 | 0.343023 |
| rs71508903 | 10 | 63779871 | T | C | 0.0143 | 0.1487 | 3.13E-25 | 108 | -0.00724 | 0.018459 | 0.694901 | 0.018973 | 0.044865 | 0.672377 |
| rs7170107 | 15 | 70010647 | T | C | 0.0158 | 0.1366 | 6.11E-18 | 75 | -0.02717 | 0.018188 | 0.135258 | -0.04671 | 0.044182 | 0.290435 |
| rs7206670 | 16 | 11833886 | T | G | 0.0119 | 0.0701 | 4.14E-09 | 35 | 0.016538 | 0.024295 | 0.496045 | -0.0433 | 0.059235 | 0.464777 |
| rs73013527 | 11 | 128496952 | T | C | 0.0128 | -0.0803 | 3.81E-10 | 39 | 0.054898 | 0.08499 | 0.518324 | -0.17933 | 0.208076 | 0.388766 |
| rs740122 | 7 | 28166442 | A | G | 0.0134 | -0.0782 | 5.37E-09 | 34 | -0.00464 | 0.020082 | 0.817326 | -0.00332 | 0.04876 | 0.945638 |
| rs76153210 | 6 | 44284508 | T | C | 0.0205 | 0.1597 | 6.83E-15 | 61 | 0.158562 | 0.153233 | 0.300773 | 0.439918 | 0.37843 | 0.245039 |
| rs7731626 | 5 | 55444683 | A | G | 0.0184 | -0.1956 | 1.94E-26 | 113 | -0.03486 | 0.067985 | 0.608155 | -0.04963 | 0.167002 | 0.766316 |
| rs7743500 | 6 | 26852053 | G | A | 0.0355 | 0.3582 | 5.98E-24 | 102 | 0.019421 | 0.033323 | 0.560023 | 0.08496 | 0.081028 | 0.294395 |
| rs7749323 | 6 | 138230389 | A | G | 0.0253 | 0.2835 | 3.47E-29 | 126 | -0.02036 | 0.019998 | 0.308682 | -0.03565 | 0.048589 | 0.463142 |
| rs77722821 | 6 | 34290482 | C | T | 0.0165 | 0.1202 | 2.78E-13 | 53 | -0.0108 | 0.023024 | 0.639153 | 0.003517 | 0.056082 | 0.949991 |
| rs8032939 | 15 | 38834033 | C | T | 0.0123 | 0.1244 | 4.47E-24 | 102 | -0.00207 | 0.018772 | 0.912189 | 0.01102 | 0.045561 | 0.808885 |
| rs8126756 | 21 | 34775444 | C | T | 0.0137 | -0.0823 | 1.81E-09 | 36 | 0.040366 | 0.03422 | 0.238158 | -0.00709 | 0.083796 | 0.9326 |
| rs8133843 | 21 | 36738242 | A | G | 0.0118 | 0.0769 | 8.78E-11 | 42 | 0.016859 | 0.018689 | 0.367013 | 0.064203 | 0.045464 | 0.157895 |
| rs9258228 | 6 | 29713613 | A | C | 0.0156 | -0.0882 | 1.59E-08 | 32 | 0.01222 | 0.018722 | 0.513948 | 0.055446 | 0.045647 | 0.2245 |
| rs9271365 | 6 | 32586794 | G | T | 0.0128 | 0.4888 | 1E-200 | 1458 | -0.01515 | 0.018681 | 0.417502 | 0.027867 | 0.045411 | 0.539437 |
| rs9405192 | 6 | 382537 | A | G | 0.0137 | -0.089 | 9.26E-11 | 42 | -0.00065 | 0.022939 | 0.977527 | 0.059566 | 0.05605 | 0.287905 |
| **Instrumental Variables of Gout** | | | | | | | | | | | | | | |
| rs3775948 | 4 | 9995182 | C | G | 0.0302 | 0.3616 | 4.81E-33 | 143 | -0.1831 | 0.0856 | 0.0324004 | 0.1005 | 0.1961 | 0.6083 |
| rs34004016 | 4 | 9697965 | C | T | 0.0278 | -0.161 | 7.06E-09 | 34 | -0.1632 | 0.0965 | 0.0909704 | 0.0208 | 0.2218 | 0.9252 |
| rs45499402 | 4 | 89043634 | C | G | 0.0509 | 0.7005 | 5.03E-43 | 189 | 0.1203 | 0.0691 | 0.0819106 | 0.0467 | 0.1582 | 0.767901 |
| rs149136965 | 6 | 30958409 | C | G | 0.0711 | 0.3958 | 2.62E-08 | 31 | 0.0152 | 0.0284 | 0.5928 | 0.0876 | 0.0651 | 0.1784 |
| rs112395288 | 19 | 50505241 | T | C | 0.0981 | 0.7483 | 2.44E-14 | 58 | -0.0221 | 0.0297 | 0.4564 | 0.0348 | 0.0683 | 0.6105 |
| rs1064257 | 19 | 49993535 | G | C | 0.0913 | 1.0269 | 2.55E-29 | 127 | 0.0127 | 0.0492 | 0.7961 | 0.0917 | 0.1141 | 0.4215 |
| rs4645896 | 19 | 49461834 | A | G | 0.0655 | 0.4085 | 4.48E-10 | 39 | -0.1139 | 0.0658 | 0.0834795 | 0.0353 | 0.1505 | 0.8144 |
