# Supplementary material for: Causal association between common rheumatic diseases and glaucoma: a Mendelian randomization study
Source: Front Immunol. 2023 Sep 19;14:1227138. doi: 10.3389/fimmu.2023.1227138 (PMC10550209; doi:10.3389/fimmu.2023.1227138)
Supplement: Supplementary file 3 [file Table_2.docx]

| Variables | Heterogeneity test | | | Pleiotropy test | |
| --- | --- | --- | --- | --- | --- |
|  | Cochrane’s Q | Qdf | pval | MR-egger pval | MR PRESSO pval |
| AS to POAG | 22.46 | 27.00 | 0.71 | 0.56 | 0.78 |
| AS to PACG | 22.46 | 27.00 | 0.71 | 0.56 | 0.13 |
|  |  |  |  |  |  |
| RA to POAG | 92.42 | 80.00 | 0.16 | 0.13 | 0.18 |
| RA to PACG | 87.80 | 80.00 | 0.26 | 0.96 | 0.20 |
|  |  |  |  |  |  |
| SLE to POAG | 41.48 | 27.00 | 0.04 | 0.10 | 0.02 |
| SLE to PACG | 27.65 | 27.00 | 0.43 | 0.16 | 0.41 |
|  |  |  |  |  |  |
| SS to POAG | 17.78 | 12.00 | 0.12 | 0.73 | 0.19 |
| SS to PACG | 8.51 | 12.00 | 0.74 | 0.08 | 0.48 |
|  |  |  |  |  |  |
| DM to POAG | 4.98 | 3.00 | 0.17 | 0.35 | 0.18 |
| DM to PACG | 0.97 | 3.00 | 0.81 | 0.71 | 0.88 |
|  |  |  |  |  |  |
| Gout to POAG | 10.71 | 5.00 | 0.06 | 0.79 | 0.15 |
| Gout to PACG | 0.90 | 5.00 | 0.97 | 0.26 | 0.90 |

***Supplementary Material***

**Genetic Causal Association between Common Rheumatic Diseases and Glaucoma: A Mendelian Randomization Study**

**Yang Meng^1†^, Zongbiao Tan^2†^, Yu Su ^1^, Lu Li ^1*^ and Changzheng Chen ^1*^**

*** Correspondence:** Lu Li (lilu-000000@163.com), Changzheng Chen ([whuchenchzh@163.com](mailto:whuchenchzh@163.com))

**Supplementary Table 2**

**Results of Cochran’s Q statistic, MR-Egger intercept test, and MR-PRESSO test**
